# Weight cycling exacerbates glucose intolerance and hepatic triglyceride storage in mice with a history of chronic high fat diet exposure

**DOI:** 10.1186/s12967-024-06039-0

**Published:** 2025-01-04

**Authors:** Miriam Bernecker, Anna Lin, Annette Feuchtinger, Anna Molenaar, Sonja C. Schriever, Paul T. Pfluger

**Affiliations:** 1 Research Unit NeuroBiology of Diabetes, Helmholtz Munich, Ingolstädter Landstraße 1, 85764 Neuherberg, Germany; 2 Institute for Diabetes and Obesity, Helmholtz Munich, Neuherberg, Germany; 3 Division of NeuroBiology of Diabetes, TUM School of Medicine & Health, Technical University of Munich, Munich, Germany; 4 German Center for Diabetes Research, Neuherberg, Germany; 5 Core Facility Pathology and Tissue Analytics, Helmholtz Munich, Neuherberg, Germany

**Keywords:** Weight cycling, Yoyo dieting, Weight loss, Obesity, Insulin resistance, Obesogenic memory

## Abstract

**Background:**

Obese subjects undergoing weight loss often fear the Yoyo dieting effect, which involves regaining or even surpassing their initial weight. To date, our understanding of such long-term obesity and weight cycling effects is still limited and often based on only short-term murine weight gain and loss studies. This study aimed to investigate the long-term impacts of weight cycling on glycemic control and metabolic health, focusing on adipose tissue, liver, and hypothalamus.

**Methods:**

Chow-fed mice and mice subjected to prolonged high-fat diet (HFD) consumption for 20 weeks, followed by 24 weeks of dietary interventions to either induce weight gain, weight loss, or weight cycling were monitored for perturbations in feeding efficiency and glucose homeostasis. Post-mortem analyses included qPCR, Western Blotting, biochemical and microscopical assessments for hepatic steatosis and insulin resistance, hypothalamic and adipose tissue inflammation, and circulating lipid, leptin and IL-6 levels.

**Results:**

Weight cycling led to hyperphagia and rapid weight regain, matching the weights of mice continuously on HFD. Despite weight loss, adipose tissue inflammation persisted with elevated pro-inflammatory markers, macrophage infiltration, and impaired Glut4 expression. HFD-induced dysregulation in hypothalamic expression of orexigenic peptides and synaptic plasticity markers persisted also after weight normalization suggesting long-lasting neural alterations. Weight-cycled mice exhibited higher circulating IL-6 and leptin levels, increased hepatic lipid storage, and dysregulated glucose metabolism compared to those with consistent diets, indicating worsened metabolic effects by Yoyo dieting.

**Conclusion:**

In sum, our study highlights significant metabolic risks associated with weight cycling, particularly following prolonged obesity. Persistent adipose tissue inflammation, perturbed neural peptide and plasticity markers and impaired glucose tolerance emphasize the need for effective and sustainable weight loss strategies to mitigate the adverse outcomes of weight regain and improve long-term metabolic health.

**Supplementary Information:**

The online version contains supplementary material available at 10.1186/s12967-024-06039-0.

## Background

More than one billion people worldwide are suffering from obesity [1], which is largely driven by our sedentary lifestyle paired with inexpensive, palatable, and highly processed foods. With its prevalence rising, this complex metabolic disorder poses a significant challenge to global health systems and increases the risk of associated diseases such as type 2 diabetes, non-alcoholic fatty liver disease (NAFLD), and metabolic syndrome [2]. The progression of comorbidities is multifactorial, yet adipocyte dysfunction and the excessive accumulation of triglycerides in the liver are common hallmarks that promote chronic low-grade inflammation, insulin resistance and glucose intolerance [3]. Fortunately, those profound metabolic alterations are mostly reversible [4, 5], and obesity-related disorders have been shown to improve already with a modest weight loss of 5% [6].

Weight loss strategies range from non-invasive interventions to highly invasive ones with increasing effectiveness. On average, individuals can lose about 5–10% of their initial body weight through dietary modifications [7], 5–20% with pharmacological interventions [8], and 20–30% or more with bariatric surgery [9, 10]. However, individuals who succeed in reducing their body weight often face an even greater struggle in maintaining this weight loss [11]. Most people regain a substantial amount of weight within 1 year, a phenomenon commonly known as weight cycling or Yoyo dieting [12, 13].

Body weight homeostasis depends on complex feedback circuits within the central nervous system (CNS) that integrate sensory and metabolic cues to orchestrate our ingestion and energy metabolism. Homeostatic centers residing within the mediobasal hypothalamus (MBH) are hereby of central importance by integrating humoral signals secreted by peripheral tissues to reflect the energy state of the body [14]. When individuals lose weight, their bodies undergo metabolic adaptations aimed at conserving energy, including a decrease in resting metabolic rate and an increase in hunger and appetite [15, 16]. Yet, the exact molecular mechanisms underlying these adaptations remain elusive [11], and rodent models of weight cycling have been developed to decipher the molecular perturbations in metabolic tissues that facilitate weight regain. Murine models of dietary weight loss thereby revealed a persistent inflammatory signature in both liver and adipose tissues, suggesting an obesogenic memory that may promote weight regain [17–19]. Further, it was demonstrated that weight cycling exacerbated appetite [20] and worsened glucose metabolism [21, 22], hepatic steatosis, and adipose tissue inflammation in comparison to animals without prior weight loss episodes [23].

However, a common limitation in many of those studies was the relatively short duration of the intervention, with intervals of 6–9 weeks of weight gain and loss by dieting [23–25]. Accordingly, less is known about systemic and organ- or tissue-specific consequences in mice subjected to a prolonged history of obesity and persistent overweight, which is more reminiscent of the human condition. In this study, we aimed to investigate the complex dynamics of weight cycling and their long-term implications on glycemic control and on the metabolic health of the adipose tissue, liver, and hypothalamus. Specifically, we subjected mice to a lengthy history of HFD consumption, followed by weight loss via transitioning from HFD to standard non-obesogenic diet and subsequent HFD-induced weight regain to monitor Yoyo dieting-driven metabolic damages and associated comorbidities. This comparison was made against mice maintained on either a life-long standard diet or HFD, or to those experiencing HFD followed by a single switch to chow diet.

## Methods

### Murine studies

AgRP-Cre mice (JAX stock #012899) were crossed with CAG-Sun1/sfGFP mice (JAX stock #021039) to obtain AgRP-Cre; Sun1sfGFP mice. Mice were housed in groups of n = 2–4 males on a 12:12 h light–dark cycle at 22 °C with free access to food (chow diet, #1314, Altromin, Germany, 3.34 kcal/g) and water. At the age of 5–10 weeks male mice were either kept on chow diet (Chow, n = 10) or switched to 58% high-fat diet (D12331, Research Diets, New Brunswick, USA, 5.56 kcal/g) for 20 weeks to induce diet-induced obesity (n = 38). HFD-fed mice were further randomly assigned into three groups: HFD (n = 13), HFD > Chow (n = 12), and Yoyo (n = 12). The HFD mice were kept on HFD for the following 24 weeks as control group for chronic obesity. HFD > Chow animals were maintained on HFD for another 12 weeks (total of 32 weeks) and then switched to chow diet (indicated by the directional >) to induce weight loss by temporary reduction of caloric intake. In contrast, the Yoyo group was switched from HFD to chow diet at study start (week 0) to induce weight loss. After having stabilized their weight in week 12, Yoyo mice were switched back to HFD to induce weight cycling. Body weight and food intake were measured weekly in grams and food intake was calculated in respect of animals per cage. Caloric intake (kcal) was calculated from dietary composition and feeding efficiency was calculated by dividing the change in body weight in grams by kcal consumed. Fat mass and lean mass were analyzed by a nuclear magnetic resonance whole-body composition analyzer (EchoMRI, TX, USA) in week 24 of the study. Animals were sacrificed ad libitum during their active phase (9:00–10:30 pm). All samples were snap frozen in liquid nitrogen and stored at − 80 °C. All animal experiments were approved by the State of Bavaria, Germany.

### HOMA-IR and glucose tolerance test

Plasma levels of glucose and insulin were measured after 8 h daytime fasting. The homeostasis model assessment of insulin resistance (HOMA-IR) value, an indicator of insulin resistance, was calculated using the following formula: [(fasting insulin [ng/mL]) × fasting glucose [mg/dL]/405] [26]. For assessment of glucose tolerance, mice were fasted for 6 h before glucose was administered intraperitoneal (i.p) at a dose of 1.5 g/kg. Tail blood glucose was measured using a handheld glucometer (FreeStyle Freedom Lite; Abbott Diabetes Care, Alameda, CA) before (0 min) and 15, 30, 60, and 120 min after the glucose injection.

### Plasma analysis

Blood samples were collected in EDTA tubes and centrifuged for 10 min at 2000xg and 4 °C. The supernatant was collected and stored at − 80 °C until further use. Plasma insulin and leptin were measured by kits from Crystal Chem, including the Ultra Sensitive Insulin ELISA Kit (#90080) and Mouse Leptin ELISA Kit (#90030). IL-6 was quantified by Mouse IL-6 Quantikine ELISA Kit from R&D systems (#M6000B). Plasma NEFA, triglyceride and cholesterol levels were determined by commercial enzymatic assay kits (Wako Chemicals, Germany;* #*434–91795 and #434–91995; #290–63701 and Thermo Scientific, #TR13421 with Cholesterol Assay Standard (10 mmol/L) from Cayman/Biozol; #CAY-10008053-1).

### Liver triglycerides

Liver triglycerides were quantified using the WAKO LabAssay Tryglyceride kit (Fujifilm Wako Diagnostics, USA, #632–50991) according to the manufacturer’s instructions.

### RNA extraction and gene expression analysis

RNA was extracted from peripheral tissues using the NucleoSpin RNA isolation kit (Macherey–Nagel, Düren, Germany) according to the manufacturer’s protocol. For RNA extraction from microdissected mediobasal hypothalamus sections, the Qiagen MicroRNeasy kit was used (#74004). Equal amounts of RNA were reverse transcribed to cDNA using the QuantiTect Reverse Transcription kit (Qiagen, Hilden, Germany). Gene expression was analyzed using custom-made primers (Sigma-Aldrich) and SYBR Green Master Mix (Applied Biosystems, Carlsbad, CA). qPCRs were carried out using a Quantstudio 6 Flex cycler (Applied Biosystems). Gene expression was evaluated using the ΔΔCt method with *Hprt* or *Rpl32* as housekeeping gene. Primer sequences to analyze glucose and lipid metabolism, ER stress and inflammation, as well as receptor and neuropeptide expression levels are listed in Suppl. Table 1.

### Western blotting and densitometric analyses

Complete RIPA lysis buffer (Sigma Life Science) with a final concentration of 1% Halt Protease & Phosphatase Inhibitor Cocktail (Thermo Fisher Scientific) and 1 mM phenylmethylsulfonyl fluoride (PMSF) were used for protein extraction. Protein concentration was measured by Pierce BCA assay (Thermo Scientific). 20 µg of protein were loaded, and size exclusion electrophoresis was done on Criterion TGX 4–20% precast gels (Bio-Rad). With a Trans Blot Turbo transfer apparatus (Bio-Rad, Hercules, CA) proteins were transferred subsequently to 0.2 μm PVDF membranes (Bio-Rad). Membranes were blocked with 5% bovine serum albumin (BSA) in tris-buffered saline with 0.1% Tween-20 (TBS-T) for 1 h at room temperature. Subsequently, membranes were incubated with anti-phospho-Insulin Receptor β Tyr1345 (p-IRβ^Tyr1345^) (#3026), anti-IRβ (#3025), anti-phospho-AKT S473 (p-AKT^S473^) (#9271), anti-AKT (#2920), anti-Fatty Acid Synthase (#3180), anti-SCD1 (#2794), anti-LPL (Santa Cruz Biotechnology; #E2814), and anti-Vinculin (1:20,000; #E1E9V) overnight at 4 °C in 5% BSA in TBS-T. All primary antibodies were purchased from Cell Signaling Technology (Danvers, MA) and diluted 1:1,000 if not indicated otherwise. After a 2 h incubation at room temperature with the secondary antibody horseradish peroxidase (HRP)-conjugated anti-rabbit IgG (1:10,000; Cell Signaling; #7074) or m-IgGκ BP-HRP (1:10,000; Santa Cruz Biotechnology; #sc-516102), membranes were detected on a ChemiDoc Imaging System (Bio-Rad) using Clarity™ Western enhanced chemiluminescence substrate (Bio-Rad, #170–5060). Densitometric quantifications were performed using Fiji ImageJ.

### Histology and immunohistochemistry

Adipose tissue specimens were fixed in neutrally-buffered 4% formaldehyde solution (Formalin 10% neutral buffered, HT501128, Sigma-Aldrich, Germany) and subsequently routinely embedded in paraffin (Tissue Tec VIP.5, Sakura Europe, Netherlands). Sections of 3 µm nominal thickness were stained with hematoxylin and eosin (H&E), using a HistoCore SPECTRA ST automated slide stainer (Leica, Germany) with prefabricated staining reagents (Histocore Spectra H&E Stain System S1, Leica, Germany), according to the manufacturer’s instructions. Immunohistochemical (IHC) detection of CD68 was performed on a Ventana Discovery Ultra-stainer (Roche Diagnostics, Germany), using specific antibodies (1:500, polyclonal rabbit anti-CD68 antibody, #125212, Abcam, USA, and secondary antibody: 1:750, goat anti-rabbit IgG antibody (H+L), biotinylated, BA-1000, Vector, Germany) and prefabricated solutions (DISCOVERY DAB Map Detection Kit, Cat. 760-124, Roche, USA).

Stained slides were digitally scanned with an AxioScan 7 scanner (Zeiss, Germany), using a 20× objective. Automated digital image analysis (Visiopharm, Hoersholm, Denmark) was used for morphometric determination of the mean adipocyte section profile areas, as well as the percentage of CD68-positive stained area per total adipose tissue section area.

### Statistical analysis

All data are presented as means ± standard error of mean (SEM). Statistical analyses were performed using GraphPad Prism 8. One-way analysis of variance (ANOVA) with Tukey’s tests were used for comparisons between multiple groups, and two-way ANOVA with Tukey’s tests used for longitudinal data. Two-tailed t-tests were run for comparisons between two groups. P < 0.05 was considered statistically significant.

## Results

### Weight cycling promotes hyperphagia and accelerates body weight regain

**Fig. 1:**
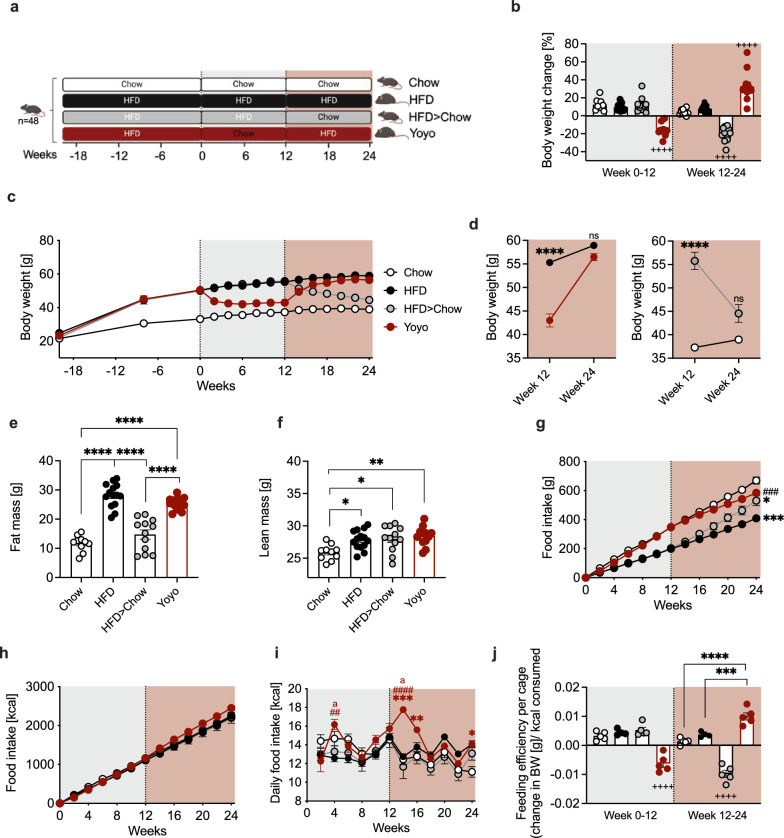
Weight loss and regain trajectories in chow and HFD-fed mice. Schematic for the study design. Yoyo mice were switched to chow diet in week 0. HFD > Chow mice were switched to chow and Yoyo mice were switched back to HFD in week 12 (**a**). Body weight changes in % in weeks 0–12 and weeks 12–24 (**b**). Body weight over time in gram (**c**). Body weight of HFD and Yoyo mice in week 12 and 24. Statistical differences between groups were determined by two-way ANOVA and Tukey’s post-hoc test. Graphs were split for better visualization (**d**). Body composition at study end, week 24 (**e**, **f**). Cumulative food intake in gram (**g**) and kcal (**h**), and daily food intake in kcal. Significance indicated for Yoyo group *p ≤ 0.05, **p ≤ 0.005, ***p ≤ 0.001 against Chow; ^#^p ≤ 0.05, ^###^p ≤ 0.001 against HFD; ^a^p ≤ 0.05 against HFD > Chow (**i**). Feeding efficiency given as change in body weight in gram divided by kcal consumed comparing weeks 0–12 and 12–24 (**j**). n = 10–14 per group. Data represented as means ± SEM. Bar graphs were analyzed using a one-way ANOVA and Tukey’s test for multiple comparisons. Two-way ANOVA and Tukey’s test was used to analyze food intake data. *p ≤ 0.05, **p ≤ 0.005, ***p ≤ 0.001, ****p ≤ 0.0001, ^++++^p ≤ 0.0001 against all other groups

Before study start, we generated a large cohort of severely obese mice by exposure to high-fat diet (HFD) for 20 weeks. Lean control animals were fed a standard chow diet in parallel and gained markedly less weight (+ 11.6 g ± 1.5 g) compared to HFD animals (+ 28.2 g ± 0.9 g). At study start, HFD-fed mice (50.1 g ± 0.9 g) were randomly assigned into three groups and followed the feeding regime as indicated in Fig. 1a. The two groups of animals kept on a continuous chow or HFD both experienced a steady weight gain of ~ 18% compared to their initial weight at week 0 (Fig. 1b, c). HFD-fed mice undergoing weight cycling were termed Yoyo mice and lost on average 14.8% (− 7.5 g ± 1.0 g) of their body weight with chow feeding from weeks 0 to 12, followed by a rapid weight regain (+ 32.9%) with HFD re-feeding from weeks 12 to 24, ultimately matching body weights of mice constantly exposed to HFD. This weight regain of 13.5 g ± 1.4 g in Yoyo mice was superior to the 4.3 g ± 0.6 g weight gain of HFD mice in the final 12 weeks of the study (Fig. 1d). Notably, despite their closely matched genetic and environmental exposures, we further observed profound interindividual variances in weight trajectories in all groups (Suppl. Figure 1). After 12 weeks of ad libitum chow-induced weight loss (− 11.2 g ± 1.5 g), HFD > Chow mice had comparable body weights as Chow mice. Fat mass reflected the nutritional status of the animals at study end, with HFD-fed animals having comparably higher fat content than chow-fed mice (Fig. 1e). Lean mass was higher in all mice previously exposed to HFD, even after the switch from HFD to chow (Fig. 1f). Dietary changes were reflected by changes in food consumption, with more grams of chow diet being consumed compared to HFD to achieve comparable kcals (Fig. 1g, h). The accelerated body weight gain seen in Yoyo mice after previous weight loss was driven by hyperphagia during the first 4 weeks on HFD (Fig. 1i) and resulted in an overall increased feeding efficiency (the ratio of weight gain to energy intake) in weeks 12–24 (Fig. 1j). Overall, the feeding efficiencies closely resembled the body weight changes.

### Moderate HFD-induced adipose tissue inflammation persists after weight loss

**Fig. 2:**
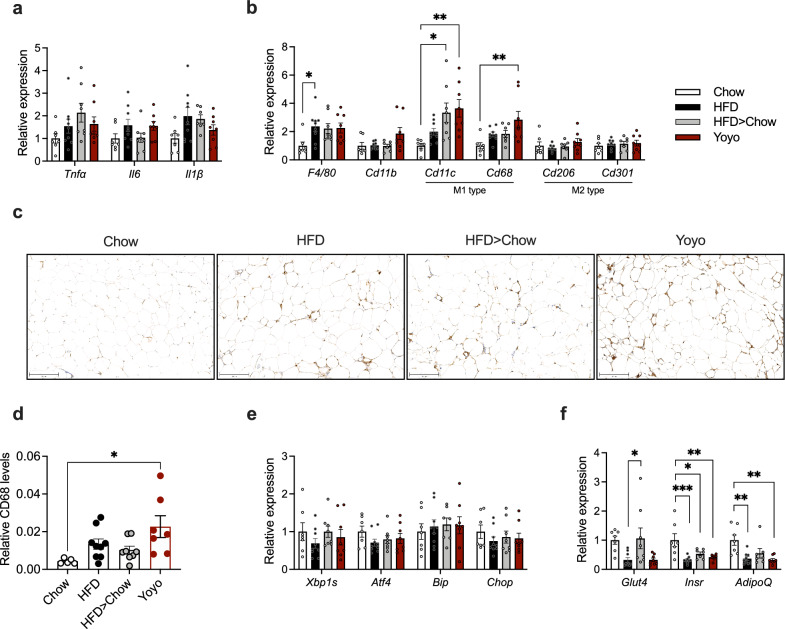
Epididymal WAT inflammation profiles. Expression levels of inflammatory (**a**), and macrophage markers (**b**). Representative CD68 stainings (**c**) and relative CD68 levels across groups, n = 5–9, scale bar = 200 µm (**d**). Expression of ER stress markers (**e**). Expression of *Glut4*, *Insr*, and *AdipoQ* in fat tissue after a 24-week dietary intervention (**f**). All genes were normalized to the housekeeping gene *Hprt*, n = 7–9. Data represented as means ± SEM. Statistical analyses were performed by one-way ANOVA and Tukey’s post-hoc multiple comparison tests; *p ≤ 0.05, **p ≤ 0.005, ***p ≤ 0.001

Adipose tissue expansion is often accompanied by a persistent inflammation and closely linked to insulin resistance [27–29]. We first compared adipocyte sizes in epididymal white adipose tissue (eWAT) of our mice but found only non-significant increases in HFD and Yoyo mice compared to the chow-fed groups (Suppl. Figure 2). Pro-inflammatory gene expression levels, such as tumor necrosis factor alpha (*Tnfa*), interleukins 6 and 1β (*Il6*, *Il1*β), as well as general markers for macrophages *F4/80* (*Emr1*) and *Cd11b* were elevated in the eWAT of HFD-fed groups (Fig. 2a, b). M1-type macrophage markers, including *Cd11c* and* Cd68* were especially increased in the Yoyo group, compared to Chow animals (Fig. 2b). Despite a reduction in body adiposity, HFD > Chow animals displayed a moderate elevation of pro-inflammatory marker genes comparable to those observed in obese animals. Immunohistochemical stainings for CD68 revealed an increased accumulation of inflammatory macrophages in Yoyo animals compared to Chow (Fig. 2c, d). The anti-inflammatory M2-type macrophage markers *Cd206* and* Cd301* remained unaltered between groups. ER-stress markers, such as spliced x-box binding protein 1 (*Xbp1s*), activating transcription factor 4 (*Atf4*), binding immunoglobulin protein (*Bip*), and C/EBP homologous protein (*Chop*), showed no significant changes across the groups (Fig. 2e). Key genes regulating glucose uptake and insulin sensitivity, namely Glucose transporter 4 (*Glut4*) and insulin receptor (*Insr*)*,* were less expressed in HFD and Yoyo mice compared to Chow animals (Fig. 2f). The weight loss group HFD > Chow showed lower levels in *Insr* expression, but similar *Glut4* levels compared to Chow animals. Expression levels of Adiponectin (*AdipoQ*), a surrogate marker for insulin sensitivity, were lower in HFD and Yoyo mice compared to lean chow mice. In sum, weight loss could not fully normalize the persistent eWAT inflammation in formerly obese mice.

### Weight cycling elevates circulating leptin levels

**Fig. 3:**
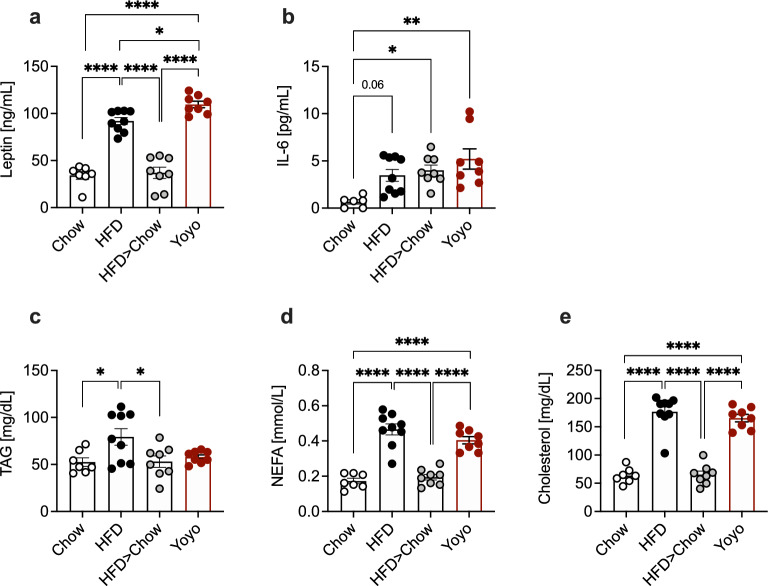
Circulating leptin, IL-6 and lipid profiles. Plasma levels of leptin (**a**), IL-6 (**b**) triacyl glycerides (TAG) (**c**), non- esterified fatty acids (NEFA) (**d**), and cholesterol (**e**) after 24 weeks of dietary interventions. Plasma levels represent the ad libitum-fed state. Data represented as means ± SEM. Statistical analyses were performed by one-way ANOVA and Tukey’s test for multiple comparisons; *p ≤ 0.05, **** ≤ 0.0001

Plasma leptin levels are highly correlated to body fat mass and circulating levels decrease and increase in response to weight loss or gain, respectively [30]. Accordingly, leptin levels resembled body fat content in our mice and weight loss normalized leptin levels in previously obese animals (Fig. 3a). Interestingly, Yoyo mice exhibited higher leptin levels than HFD animals despite comparable fat mass. IL-6 levels were elevated in both, the obese and formerly obese mice compared to Chow animals (Fig. 3b). Plasma triacyl glycerides (TAGs), non- esterified fatty acids (NEFAs), and cholesterol content reflected dietary fat intake, with overall increased levels in the HFD- compared to chow-fed animals (Fig. 3c–e).

### Yoyo dieting increases hepatic lipid storage

**Fig. 4:**
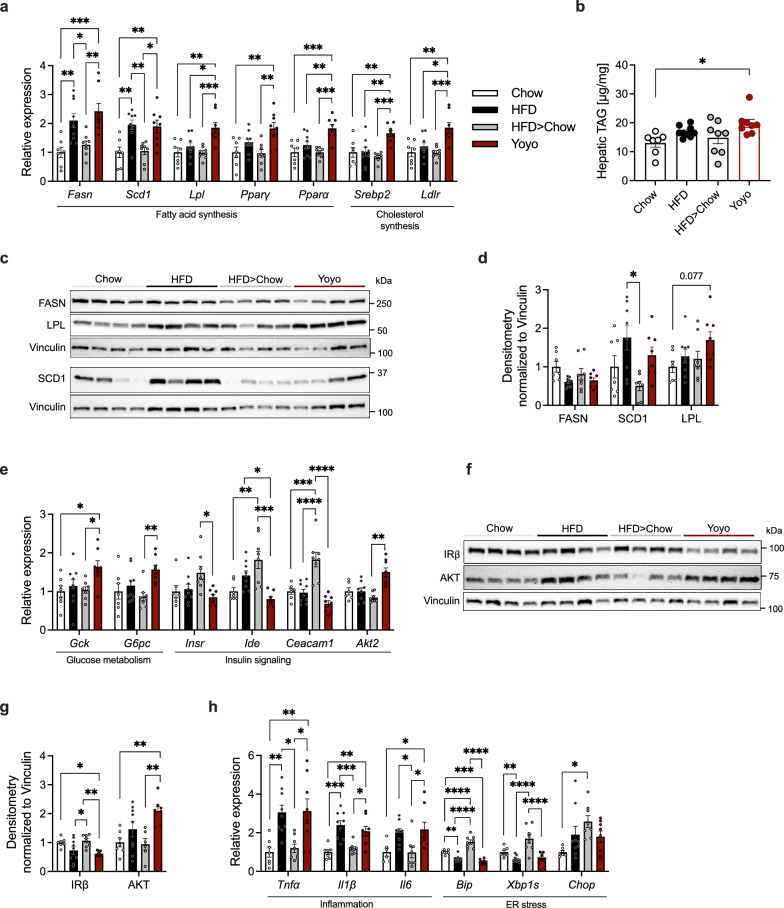
Changes of hepatic lipid and cholesterol metabolism. Expression changes for key enzymes involved in fatty acid and cholesterol metabolism (**a**). Hepatic TAG levels, n = 6–8 (**b**). Representative western blot image (**c**) and densitometric quantification of FASN, SCD1, and LPL protein levels relative to the housekeeper Vinculin, n = 7–9 (**d**). mRNA levels of genes involved in glucose metabolism and insulin turnover (**e**). Representative western blot image (**f**) and densitometric quantification of IRβ and AKT relative to Vinculin, n = 7–9 (**g**). Gene expression of inflammatory and ER stress markers (**h**). Gene expression was normalized to the housekeeping gene *Rpl32*, n = 7–9. Data represented as means ± SEM. Statistical analyses were performed by one-way ANOVA and Tukey’s post-hoc tests for multiple comparisons; *p ≤ 0.05, **p ≤ 0.005, ***p ≤ 0.001, ****p ≤ 0.0001

We next assessed the impact of weight loss and weight cycling on the expression of hepatic mRNA and proteins involved in lipid and glucose metabolism using quantitative real-time PCR and Western Blot analyses. Fatty acid synthase (*Fasn*) and stearoyl-coenzyme A desaturase 1 (*Scd1*) were substantially increased in both HFD and Yoyo animals. In contrast, additional genes involved in fatty acid and cholesterol synthesis such as lipoprotein lipase (*Lpl*), peroxisome proliferator-activated receptor (*Ppar*) α and γ, serpine1 mRNA binding protein 2 (*Srebp2*) and low-density lipoprotein receptor (*Ldlr*) were only elevated in Yoyo mice, compared to HFD, HFD > Chow and Chow animals (Fig. 4a). In agreement with this, weight cycled Yoyo mice had significantly (p = 0.0146) elevated hepatic TAG levels compared to Chow mice (Fig. 4b), pointing toward increased hepatic lipid accumulation due to weight cycling. Hepatic protein levels of FASN nonetheless remained unchanged across all groups, while SCD1 levels were elevated in HFD-fed compared to HFD > Chow mice. LPL protein levels followed the trend observed in qPCR analyses, with a slight increase in Yoyo compared to Chow mice (Fig. 4c, d).

Elevated levels of both glucokinase (*Gck*) and glucose-6-phosphatase (*G6pc*) in the livers of Yoyo mice indicated a dysregulation in hepatic glucose metabolism, with increased glucose uptake and utilization (*Gck*) and increased glucose production (*G6pc*). Insulin-degrading enzyme (*Ide*) as well as carcinoembryonic antigen-related cell adhesion molecule 1 (*Caecam1*) were elevated in HFD > Chow compared to all other groups (Fig. 4e). Decreased mRNA and protein levels of insulin receptor β (qPCR: *Insr*; protein: IRβ) and increased Akt2 mRNA and total AKT protein levels in the Yoyo group, compared to Chow and/or HFD > Chow mice (Fig. 4e, f, g) are further highlighting an impaired glucose metabolism and insulin resistance after weight cycling. In contrast, phosphorylated IRβ and AKT levels, as well as their respective ratios to total protein, were comparable across all groups, likely attributable to the absence of an acute insulin stimulus (Suppl. Figure 3). Weight loss animals showed no further improvements in genes related to glucose metabolism as compared to Chow animals, but they did show alterations in insulin signaling (Fig. 4e). In addition to exhibiting comparable IRβ and AKT levels to Chow mice (Fig. 4f, g), these findings suggest improved insulin sensitivity following weight loss.

HFD feeding increased inflammatory gene markers in the livers of HFD and Yoyo mice (Fig. 4h), as previously reported [31]. Weight loss normalized expression levels of *Tnfa*, *Il6,* and *Il1β* (Fig. 4h). Notably, the weight loss group showed a slight increase in ER stress markers *Bip* and *Xbp1s* compared to the other groups and an increase in *Chop* when comparing to the Chow group. Diminished levels of ER stress markers *Bip* and *Xbp1s* in HFD and Yoyo mice, compared to Chow and HFD > Chow animals, are furthermore reminiscent with resolution of, or failure to adequately respond to ongoing ER stress in the liver. Overall, dietary weight loss was associated with alleviated metabolic dysregulations of lipid metabolism, diminished inflammation, and improved glucose metabolism, whereas weight cycling tended to worsen the metabolic effects of HFD feeding on the liver.

### Weight cycling exacerbates HFD-induced glucose intolerance and insulin resistance

**Fig. 5:**
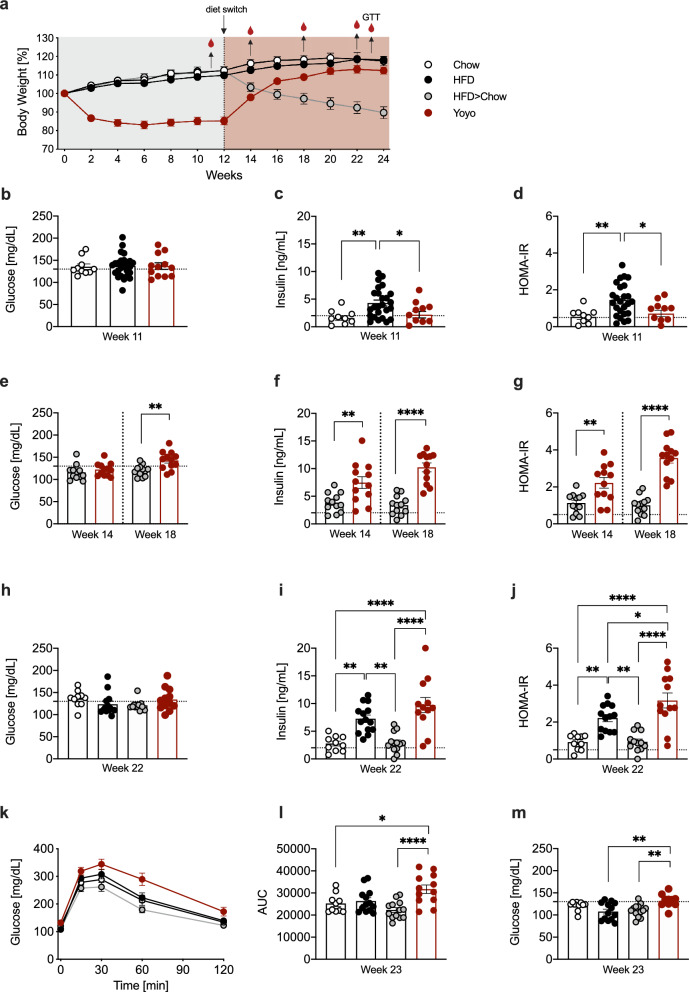
Weight cycling perturbs glucose metabolism. Body weight over time in %. Diet switch, blood withdrawals, and GTT are indicated with black arrows (**a**). 8 h fasting glucose 1 week before diet switch, week 11, dotted line indicates 130 mg/dl; plasma insulin levels, dotted line indicates 2 ng/ml and HOMA-IR, dotted line indicates 0.5 (**b**–**d**). 8 h fasting glucose, plasma insulin levels and HOMA-IR two and 6 weeks after diet switch, week 14 and 18 (**e**–**g**). 8 h fasting glucose, plasma insulin levels and HOMA-IR 10 weeks after diet switch, week 22 (**h**–**j**). Intraperitoneal glucose tolerance test (GTT) (**k**), Area Under the Curve (**l**; AUC), and 6 h fasting levels of blood glucose (**m**), week 23, n = 10–13. Data represented as means ± SEM. Statistical analyses were performed by one-way ANOVA and Tukey’s test for multiple comparisons, and by two-tailed unpaired t-tests when comparing two groups; *p ≤ 0.05, **p ≤ 0.005, ***p ≤ 0.001, ****p ≤ 0.0001

To assess systemic metabolic consequences, including the state of insulin resistance during critical periods of weight loss and regain in our study, we collected several blood samples (Fig. 5a) during the 24 weeks of dietary intervention and estimated insulin resistance by homeostatic model assessment of insulin resistance (HOMA-IR). These values correlated positively with body weights in both Yoyo (r = 0.6106) and HFD > Chow (r = 0.7233) animals (Suppl. Figure 4).

Following 11 weeks of ad libitum chow diet, Yoyo mice had lost ~ 15% of their body weight. While fasting blood glucose levels were similar between all groups (~ 130 mg/dl) (Fig. 5b), Yoyo mice exhibited improved insulin sensitivity compared to the HFD-fed mice, as reflected by decreased insulin and HOMA-IR levels (Fig. 5c, d). Subsequent HFD re-feeding at week 12 significantly increased blood glucose, insulin and HOMA-IR levels in Yoyo mice compared to HFD > Chow animals despite comparable body weights (HOMA-IR week 14, 2.2 ± 0.3 vs. 1.1 ± 0.2, p = 0.0029; Fig. 5e, f, g). At week 22, Yoyo mice exhibited similar glucose and insulin levels, but elevated HOMA-IR values compared to weight matched HFD mice (Fig. 5h, i, j). A GTT at week 23 confirmed impaired glucose tolerance in Yoyo compared to chow-fed mice (Fig. 5k, l). Fasting glucose levels were moreover elevated in the Yoyo group compared to HFD and HFD > Chow animals (Fig. 5m). Notably, glucose tolerance was similar between HFD and control Chow animals, indicating an age-related (48 ± 5 weeks) decline in glucose tolerance.

### Persistent hypothalamic gene expression changes in mice with a history of HFD exposure

**Fig. 6:**
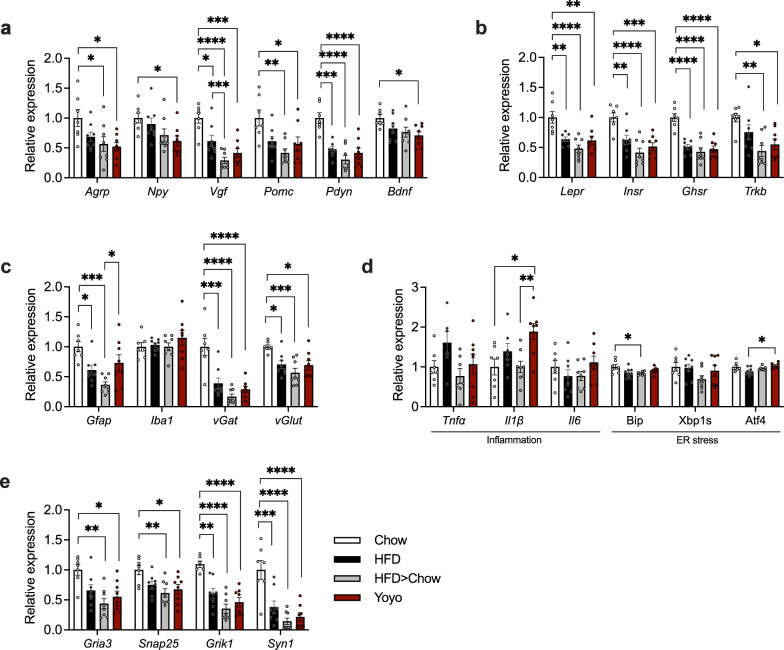
Current and past HFD exposure perturbs hypothalamic gene expression. Expression levels of orexigenic and anorexigenic genes (**a**) and hormone receptors (**b**) in the mediobasal hypothalamus. Gene markers for glial cells, GABAergic, and glutamatergic neurons (**c**). Gene expression for markers of inflammation and ER stress (**d**) as well as synaptic function and plasticity (**e**). All genes were normalized to the housekeeping gene *Hprt*, n = 7–9. Data represented as means ± SEM. Statistical analyses were performed by one-way ANOVA and Tukey’s test for multiple comparisons; *p ≤ 0.05, **p ≤ 0.005, ***p ≤ 0.001, ****p ≤ 0.0001

The mediobasal hypothalamus (MBH) plays a central role in orchestrating feeding behavior. We found diminished expression levels of orexigenic and anorexigenic neuropeptides (Fig. 6a) and receptors for leptin, insulin, ghrelin and brain-derived neurotrophic factor (BDNF) (Fig. 6b) in the MBH of HFD, Yoyo, and weight loss mice compared to Chow animals. Astrocyte marker glial fibrillary acidic protein (*Gfap*) mRNA levels were lower in HFD and HFD > Chow mice compared to Chow, and in HFD > Chow compared to Yoyo mice. Ionized calcium binding adapter molecule 1 (*Iba1*), a marker for microglial cells, showed no alterations between groups. Expression levels for the GABAergic marker *vGat* and glutamatergic marker *vGlut* were diminished in all groups compared to Chow (Fig. 6c). Inflammatory and ER stress markers were largely unaffected except for increased *Il1β* levels in Yoyo compared to Chow and HFD > Chow mice, diminished *Bip* in HFD > Chow compared to Chow, and increased *Atf4* levels in Yoyo compared to HFD mice (Fig. 6d). Notably, despite their weight loss of ~ 11% in the 12 weeks following the dietary switch, HFD > Chow animals displayed mRNA levels for marker genes of synaptic function and plasticity that resembled those of the HFD animals (Fig. 6e). Overall, largely comparable gene regulations between all HFD- and formerly HFD-fed mice suggested a persistent dysregulation caused by HFD feeding that couldn’t be reversed with weight loss.

## Discussion

“The dose makes the poison” is an adage particularly relevant when considering the metabolic consequences of HFD exposure. With our paradigm of 20 weeks of HFD feeding, we aimed to provide insights into the potential health risks associated with a prolonged “dose” of HFD. Subsequent dietary interventions were applied for 24 weeks, with weight loss being induced by a dietary switch from HFD to ad libitum standard chow to avoid the involuntary restriction of calories often observed in typical fasting studies [32]. We hereby aimed to delineate whether metabolic perturbations by chronic HFD exposure are permanent, reversible via weight loss, and exacerbated by weight cycling.

HFD-induced obesity is known to increase inflammatory gene expression in the adipose tissue [33]. In the eWAT of our HFD-fed mice, only mRNA levels of macrophage marker F4/80 were elevated. This increase was also present in Yoyo mice, which also showed higher levels of macrophages confirmed by immunohistochemical staining. This mild inflammation also persisted to some extent after weight loss, consistent with a purported “obese fingerprint [17–19] and an innate memory in adipose macrophages enhancing inflammation upon weight regain [24].

Malfunctioning adipocytes have been associated with an impaired lipid storage capacity, leading to compensatory fat deposition in the liver and metabolic dysfunction-associated fatty liver disease (MAFLD) [39]. This is consistent with elevated triglyceride levels in the livers of our Yoyo mice and higher mRNA and protein levels of lipogenic enzymes. Weight loss in the HFD > Chow mice restored expression levels of lipogenic enzymes to the levels of Chow mice. We likewise found comparable low mRNA levels of hepatic inflammatory genes in Chow and HFD > Chow mice, and increased levels in HFD and Yoyo mice. Surprisingly, however, Chow and especially HFD > Chow mice showed inconsistent levels of ER stress markers when compared to HFD and Yoyo mice. Previous research in both humans and rodents has documented an increase in ER stress with HFD feeding and a relief upon weight loss [35–37]. These reports contrast with our study and elevated Chop, but diminished Bip and Xbp1s levels in HFD-fed mice, potentially indicating different trajectories and a major influence of exogenous, environmental factors in the etiologies of hepatic inflammation, ER stress and MASLD in mice with stable obesity versus those undergoing weight fluctuations.

Elevated immune responses after weight cycling, such as the elevated circulating IL-6 levels in our Yoyo mice, were reported to associate with an impaired systemic glucose tolerance and insulin sensitivity [34, 35]. Accordingly, in our Yoyo mice we observed a significant deterioration in insulin sensitivity, estimated via HOMA-IR assessments, already 2 weeks after HFD re-feeding compared to weight-matched mice undergoing weight loss. This decline in insulin sensitivity worsened with additional weeks of HFD re-feeding and even exceeded the values observed in animals maintained on a chronic HFD regimen. Lower insulin receptor beta (IRβ) and elevated glucoregulatory Gck, G6pc or Akt levels particularly in Yoyo mice were consistent with such detrimental effects of weight cycling. Overall, our data highlight the dilemma of Yoyo dieting and point toward an obesogenic memory upon HFD re-exposure that exacerbates systemic glucose intolerance and tissue-specific metabolic pathologies.

The concept of an obesogenic memory that contributes to the Yoyo weight regain effect is intriguing, but the exact nature for that phenomenon and its tissue-of-origin are highly debated. In formerly obese human patients, adipose tissue was shown to retain an epigenetic memory of obesity even 2 years after surgical weight loss [36]. This finding was confirmed in mice and moreover linked to an accelerated rebound weight gain when mice were re-exposed to a HFD [36]. Molecular mechanisms for that rebound effect remained elusive but likely entail crosstalk to other metabolic organs such as the liver, muscle or the CNS with its homeostatic centers in the brain stem or hypothalamus. After calorie restriction, mice reportedly respond with hyperphagia [37, 38] and rapid weight regain [20], shown here as elevated caloric intake persisting for 4 weeks in Yoyo mice compared to HFD controls. Yoyo mice nonetheless didn’t overshoot in body weight despite an overall increased food consumption.

A prominent peripheral signal implicated in the CNS control of food intake and body weight that differed between Yoyo and HFD mice was the adipocytokine hormone leptin. In obese individuals, circulating leptin levels are elevated [30], but fail to decrease appetite and food intake due to a phenomenon termed leptin resistance [39]*.* Elevated leptin levels in HFD-fed obese mice were moreover attributed with detrimental health outcomes and the genetic and pharmacological removal of such leptin levels was sufficient to improve metabolic health in mice [40]. Prompted by our finding of increased mRNA levels of the inflammatory leptin-target Il1b [41] in the hypothalamus, it is thus tempting to speculate that the elevated leptin levels in our Yoyo mice may drive metabolic impairments such as insulin resistance, potentially via direct effects on glucoregulatory hypothalamic LepR-positive neurons. Here, orexigenic AgRP neurons and anorexigenic POMC neurons residing in the ARC of the hypothalamus are of special importance, as they orchestrate the homeostatic control of metabolism by integrating hormonal signals such as leptin [41]. When animals are exposed to highly palatable foods, AgRP neurons were shown to desensitize not only to leptin [42], potentially as a protective mechanism against hyperleptinemia, but also to less desirable food low in fat and sugar, such as chow. Together with alterations in hedonic feeding responses, this may promote binge-eating behaviors [43] consistent with the overfeeding we observed in our mice.

In line with the inhibitory role of leptin in controlling the expression of AgRP and NPY [44, 45], many studies have shown that HFD feeding, and the associated hyperleptinemic state, can reduce the expression of orexigenic peptides in the ARC of obese mice [25, 46, 47]. We confirm the tendency of reduced expression levels of orexigenic peptides, including *Agrp*, *Npy* and *Vgf* in the MBH of chronic HFD and weight-cycled mice, indicating decreased appetite. As reported before [46], *Pomc* levels were reduced in HFD-fed mice despite the abundance of leptin. This may be a consequence of an impaired leptin responsiveness as shown by others [48], or an overall lower expression of Lepr levels compared to Chow as observed in our experiment. Receptors for insulin, ghrelin and BDNF showed similarly reduced gene expression levels. Intriguingly, HFD > Chow animals exhibited similar gene expression levels as the HFD and Yoyo mice despite weight normalization. This not only encompassed genes encoding receptors but also neuropeptides, highlighting that a history of long-term obesity may induce non-reversible changes in the brain. Supporting this notion, synaptic plasticity markers showed comparable expression levels in both the weight loss and HFD groups, and the overall reduction in expression indicated a significant impairment in plasticity compared to the control Chow condition.

## Conclusion

In summary, our study highlights the metabolic risks for individuals undergoing weight cycling, especially after long-lasting obesity. Many obesity-induced metabolic perturbations of prolonged obesity and 32 weeks of HFD appear to remain reversible by normalizing body weight, especially in the liver. However, others, particularly in the adipose tissue, may never fully recover, or may require a longer weight stabilization period. For instance, even 2 years after bariatric surgery, formerly obese patients still retain an epigenetic memory of obesity in white adipose tissue that may promote exacerbated weight re-gain in a hypercaloric environment. Future studies will need to clarify how long such an obesogenic memory in adipose tissue persists, and whether it indeed is causally linked to the higher propensity for weight regain. The recent development of efficient weight loss drugs based on Glucagon-like Peptide 1 (GLP-1) and Gastric inhibitory polypeptide (GIP) agonism such as semaglutide and tirzepatide makes that question even more urgent; patients profoundly lose weight but suffer from weight regain as soon as the drugs are stopped [49]. Especially for this growing group of weight loss patients, it will be imperative to fully delineate, and prevent, the molecular mechanisms of Yoyo dieting and its deleterious effect on glucose homeostasis compared to weight matched obese controls. Although our study did not identify exact molecular causes for the impaired glucose control in the Yoyo mice, our data on persistent adipose tissue inflammation signals and deregulated neuropeptide profiles nonetheless suggest impaired communication signals between adipose tissue and the brain. Whether the adipocytokine leptin, which was elevated in the Yoyo mice, could play a major role in this phenomenon as a likely culprit, remains to be investigated. Such studies should further identify the specific neuronal populations affected to fully understand the mechanisms behind the accelerated body weight gain observed. The hypothalamus, as a major site of weight loss-induced perturbations, may be the prime suspect and primary focus to unravel these mechanisms comprehensively.

## Supplementary Information

Supplementary Material 1.

Supplementary Material 2. Suppl. Figure 1. Individual weight trajectories across all groups. The extent of weight loss and gain in % of the starting BW at week 0 varies profoundly in each individual animal of the Chow; n=10, HFD; n=13, HFD>Chow; n=12, and Yoyo; n=12groups. Each line represents an individual animal

Supplementary Material 3. Suppl. Figure 2. Adipocyte size. Representative image of H&E-stained eWAT sectionsand adipocyte size across all groups, n=7-9, scale bar= 200 µm. Data represented as means ± SEM. Statistical analyses were performed by one-way ANOVA and Tukey’s test for multiple comparisons

Supplementary Material 4. Suppl. Figure 3. Hepatic insulin signaling. Representative western blot imagesand densitometric quantification of phosphorylated IRβ and AKT relative to Vinculinand total protein, n=7-9. Data represented as means ± SEM. Statistical analyses were performed by one-way ANOVA and Tukey’s test for multiple comparisons.

Supplementary Material 5. Suppl. Figure 4. HOMA-IR correlates positively with body weight. Table of HOMA-IR ± SEM of all 4 groups across weeks 11-22 (a). Correlation of body weight and HOMA-IR in HFD>Chow (n=12) (b) and Yoyo (n=12) mice (c)

## Data Availability

All data generated or analyzed during this study are included in this article, and its supplementary information files. The datasets underlying this article are available on reasonable request to the corresponding author.

## Abbreviations

AdipoQ: Adiponectin
Agrp: Agouti related peptide
Akt: Serine/threonine-protein kinase (Protein Kinase B)
Atf4: Activating transcription factor 4
Bdnf: Brain-derived neurotrophic factor
Bip: Binding immunoglobulin protein
Caecam1: Carcinoembryonic antigen-related cell adhesion molecule 1
Cd11b: Integrin Subunit Alpha M
Cd11c: Integrin Subunit Alpha X
Cd206: Mannose Receptor C-Type 1
Cd301: C-Type Lectin Domain Family 10, member A
Cd68: Cluster of Differentiation 68
Chop: C/EBP homologous protein
CNS: Central nervous system
F4/80: Adhesion G Protein-Coupled Receptor E1
Fasn: Fatty acid synthase
G6pc: Glucose-6-phosphatase
Gck: Glucokinase
Gfap: Glial fibrillary acidic protein
Ghsr: Growth Hormone Secretagogue Receptor
GIP: Gastric inhibitory polypeptide
GLP-1: Glucagon-like Peptide 1
Glut4: Glucose Transporter 4
Gria3: Glutamate Ionotropic Receptor AMPA Type Subunit 3
Grik1: Glutamate Ionotropic Receptor Kainate Type Subunit 1
H&E: Hematoxylin and eosin
HFD: High fat diet
HOMA-IR: Homeostasis model assessment of insulin resistance
Hprt: Hypoxanthine phosphoribosyltransferase
i.p: Intraperitoneal
Iba1: Ionized calcium binding adapter molecule 1
Ide: Insulin-degrading enzyme
II1β: Interleukin 1β
Il6: Interleukin 6
Insr: Insulin receptor
Ldlr: Low-density lipoprotein receptor
Leprb: Leptin receptor b
Lpl: Lipoprotein lipase
MAFLD: Metabolic associated fatty liver disease
MBH: Mediobasal hypothalamus
NAFLD: Non-alcoholic fatty liver disease
NEFA: Non- esterified fatty acid
Npy: Neuropeptide Y
Pdyn: Prodynorphin
Pomc: Proopiomelanocortin
Pparα: Peroxisome proliferator-activated receptor α
Pparγ: Peroxisome proliferator-activated receptor γ
Rpl32: Ribosomal protein L32
Scd1: Stearoyl-coenzyme A desaturase 1
SEM: Standard error of mean
Snap25: Synaptosome associated protein 25
Srebp2: Serpine1 mRNA binding protein 2
Syn1: Synapsin I
TAG: Triacyl glyceride
Tnfa: Tumor necrosis factor alpha
Trkb: Tropomyosin Receptor Kinase B
vGat: Vesicular GABA Transporter, Slc32a1
Vgf: VGF Nerve Growth Factor Inducible
vGlut2: Vesicular Glutamate Transporter, Slc17a6
Xbp1s: X-Box Binding Protein 1, spliced form
