# Supplementary figures and images for: Weight cycling exacerbates glucose intolerance and hepatic triglyceride storage in mice with a history of chronic high fat diet exposure
Source: J Transl Med. 2025 Jan 4;23:7. doi: 10.1186/s12967-024-06039-0 (PMC11699648; doi:10.1186/s12967-024-06039-0)

**a**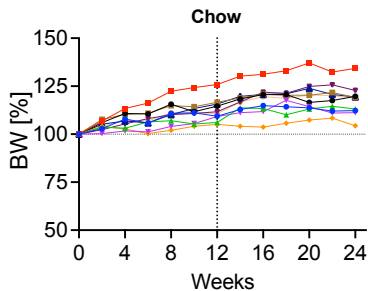**b**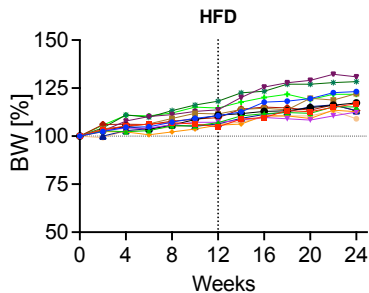**c**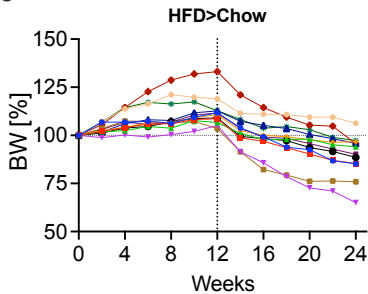**d**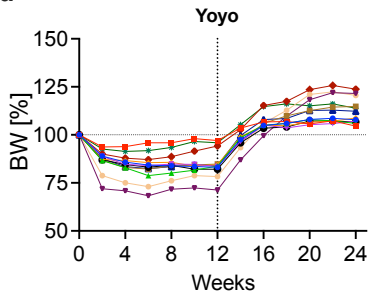

Supplement: Supplementary file 2 — Supplementary Material 2. Suppl. Figure 1. Individual weight trajectories across all groups. The extent of weight loss and gain in % of the starting BW at week 0 varies profoundly in each individual animal of the Chow; n=10, HFD; n=13, HFD>Chow; n=12, and Yoyo; n=12groups. Each line represents an individual animal [file 12967_2024_6039_MOESM2_ESM.pdf]

**a**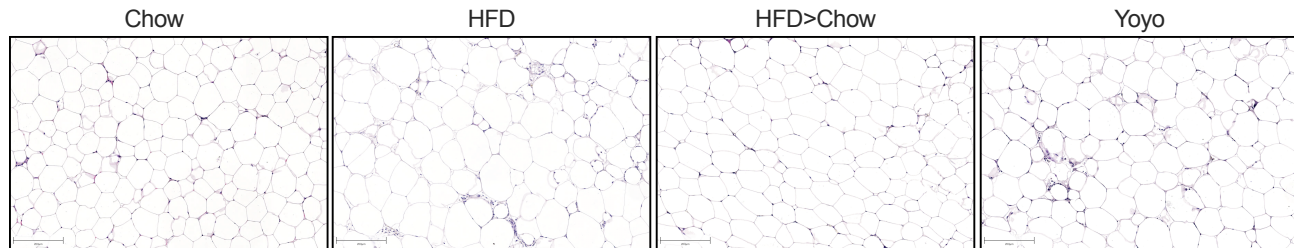**b**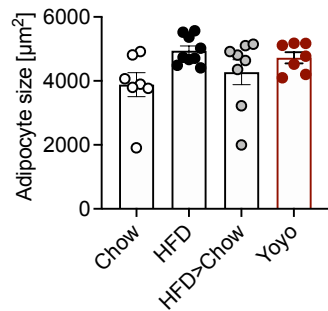

Supplement: Supplementary file 3 — Supplementary Material 3. Suppl. Figure 2. Adipocyte size. Representative image of H&E-stained eWAT sectionsand adipocyte size across all groups, n=7-9, scale bar= 200 µm. Data represented as means ± SEM. Statistical analyses were performed by one-way ANOVA and Tukey’s test for multiple comparisons [file 12967_2024_6039_MOESM3_ESM.pdf]

**a**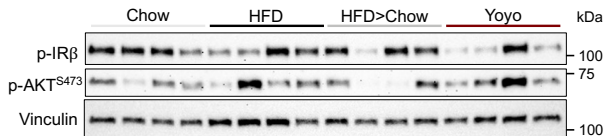**b**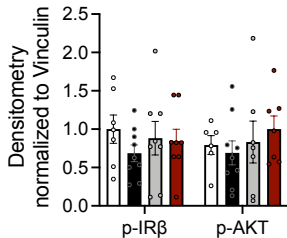**c**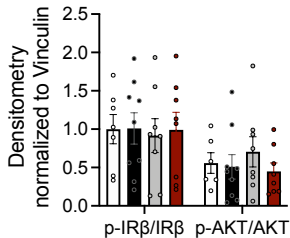

Supplement: Supplementary file 4 — Supplementary Material 4. Suppl. Figure 3. Hepatic insulin signaling. Representative western blot imagesand densitometric quantification of phosphorylated IRβ and AKT relative to Vinculinand total protein, n=7-9. Data represented as means ± SEM. Statistical analyses were performed by one-way ANOVA and Tukey’s test for multiple comparisons. [file 12967_2024_6039_MOESM4_ESM.pdf]

**a**

HOMA-IR

|          | <b>week 11</b> | <b>week 14</b> | <b>week 18</b> | <b>week 22</b> |
|----------|----------------|----------------|----------------|----------------|
| Chow     | 0.5 ±0.1       |                |                | 0.9 ±0.1       |
| HFD      | 1.4 ±0.2       |                |                | 2.2 ±0.2       |
| HFD>Chow |                | 1.1 ±0.2       | 1.0 ±0.1       | 0.9 ±0.2       |
| Yoyo     | 0.7 ±0.2       | 2.2 ±0.3       | 3.6 ±0.3       | 3.2 ±0.4       |

**b****HFD>Chow**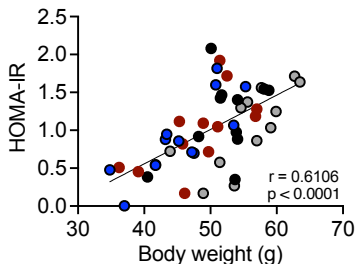**c****Yoyo**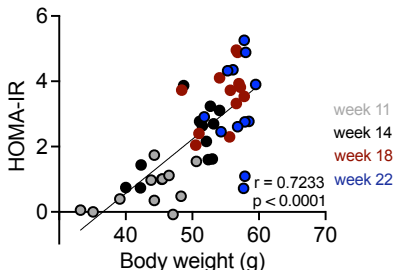

Supplement: Supplementary file 5 — Supplementary Material 5. Suppl. Figure 4. HOMA-IR correlates positively with body weight. Table of HOMA-IR ± SEM of all 4 groups across weeks 11-22 (a). Correlation of body weight and HOMA-IR in HFD>Chow (n=12) (b) and Yoyo (n=12) mice (c) [file 12967_2024_6039_MOESM5_ESM.pdf]
